# Evidence for germline non-genetic inheritance of human phenotypes and diseases

**DOI:** 10.1186/s13148-020-00929-y

**Published:** 2020-09-11

**Authors:** Liana Senaldi, Matthew Smith-Raska

**Affiliations:** 1 Division of Newborn Medicine, Department of Pediatrics, Weill Cornell Medicine, New York-Presbyterian Hospital, New York, NY USA; 2 Drukier Institute for Children’s Health, Weill Cornell Medicine, New York, NY USA

**Keywords:** Human epigenetic inheritance, Human disease inheritance, Transgenerational epigenetic inheritance, Sperm DNA methylation, Sperm small RNAs, Thyroid hormone epigenetics, Reduced sensitivity to thyroid hormone, Överkalix study, Parent-of-origin

## Abstract

It is becoming increasingly apparent that certain phenotypes are inherited across generations independent of the information contained in the DNA sequence, by factors in germ cells that remain largely uncharacterized. As evidence for germline non-genetic inheritance of phenotypes and diseases continues to grow in model organisms, there are fewer reports of this phenomenon in humans, due to a variety of complications in evaluating this mechanism of inheritance in humans. This review summarizes the evidence for germline-based non-genetic inheritance in humans, as well as the significant challenges and important caveats that must be considered when evaluating this process in human populations. Most reports of this process evaluate the association of a lifetime exposure in ancestors with changes in DNA methylation or small RNA expression in germ cells, as well as the association between ancestral experiences and the inheritance of a phenotype in descendants, down to great-grandchildren in some cases. Collectively, these studies provide evidence that phenotypes can be inherited in a DNA-independent manner; the extent to which this process contributes to disease development, as well as the cellular and molecular regulation of this process, remain largely undefined.

## Introduction

The traditional Mendelian model of inheritance states that phenotypes are inherited based on the transmission of DNA sequences across generations, and diseases are inherited when these DNA sequences are abnormal. In model organisms, it is becoming increasingly apparent that phenotypes and disease risk can be inherited from sperm and oocytes in the absence of DNA mutations or variations. This non-genetic inheritance is based on the concept that non-DNA molecules in sperm and oocytes are inherited at fertilization and modify the phenotype of the offspring, sometimes across multiple generations. The central concept in this field is that an organism’s exposures (diet, stress, chemicals, etc.) affect the composition of germline non-DNA molecules, and in this manner, these exposures can affect phenotypes in descendants. Localization of this phenomenon to the germ cells is proven by the use of in vitro fertilization with surrogate “mothers” carrying the fetus who never experienced the exposure [1]. While there are multiple examples of this phenomenon across a variety of model organisms [1–4], the role of this process in human inheritance is less well described, and the cellular and molecular mechanisms that drive the non-genetic inheritance of phenotypes across generations are similarly poorly characterized.

This process is often referred to as “epigenetic inheritance.” Epigenetics refers to the modification of a phenotype without a change to the DNA sequence itself, and epigenetic factors are specific molecules that affect gene expression without changing the DNA sequence; examples include DNA methylation and chromatin modifications. In sperm and oocytes, these epigenetic molecules can be inherited at fertilization and thereby affect fetal organ development by modifying patterns of gene expression. These germline epigenetic factors are potentially altered based on an individual’s experiences and exposures, and in this manner, epigenetic abnormalities in sperm and oocytes can have significant effects on a descendant’s risk of disease. The germline factors discussed here include DNA methylation and small RNAs. DNA methylation is the covalent addition of a methyl group to DNA, which often leads to gene silencing [5]. Small RNAs are a diverse group of molecules that do not code for protein; rather they have diverse (and in some cases, poorly understood) functions including the modulation of gene expression. Small RNAs are not traditional epigenetic molecules because they mostly target RNA molecules rather than DNA, and are therefore more accurately described as post-transcriptional gene expression regulators. Examples of small RNAs include miRNA, piRNA, snoRNA, and tRNA-derived fragments [6]. Histone modifications are another common epigenetic modification which is not discussed in this review because their role in germline-mediated inheritance is largely undescribed, due to the epigenetic reprogramming that characterizes germ cell development (see below). While epigenetic factors are important contributors to inherited phenotypes caused by ancestral exposures in model organisms [1, 7], their role in human exposure-driven inheritance is undescribed; thus, we use the term “non-genetic inheritance” to describe this process in humans.

This review focuses on examples of germline-mediated non-genetic inheritance that have been described in humans. There are many excellent reviews of this process in other organisms, which will not be discussed here [8–10]. The existence of non-genetic germline inheritance in humans is a controversial topic for a number of reasons, especially due to the confounding factors encountered when measuring human phenotypes across generations, which makes the critical evaluation of findings especially important [11].

### General approaches to detect germline-mediated non-genetic inheritance in humans

**Fig. 1:**
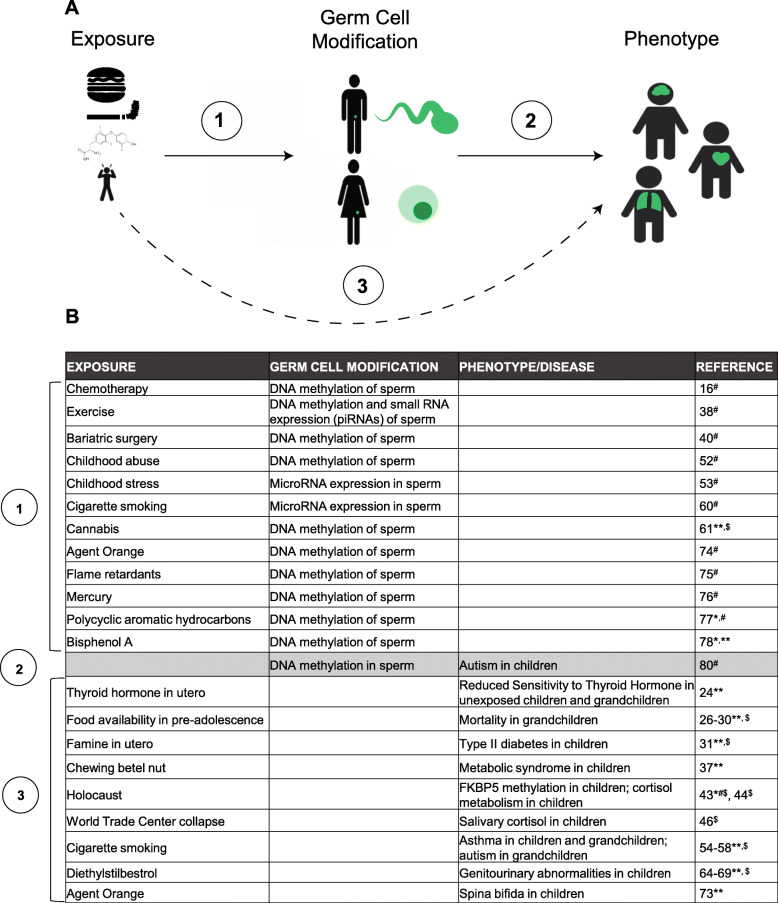
Approach to Connecting Ancestral Exposures to Descendants’ Phenotypes Through Germ Cell Modifications. **a** In humans, no single study has demonstrated a connection in which an exposure leads to a germ cell modification, ultimately manifesting as a disease or phenotype in descendants. Various exposures (food, cigarette smoking, in utero thyroid hormone exposure, stress, etc.) have been associated with changes in germ cell epigenetic processes (1); exposures have also been associated with diseases in children and grandchildren (3). Less commonly, a disease has been associated with germ cell abnormalities in anindividual’s parents (2). **b** Table summarizing specific findings regarding the associations described in (**a**).

One of the most powerful approaches to detect non-genetic inheritance through the germline is the observation of a phenotype in descendants after a well-defined exposure in an ancestor. Importantly, this approach does not exclude the contribution of non-germline factors to inheritance, such as the role of social or genetic processes in phenotype inheritance. To our knowledge, no single human study has drawn a clear connection from (1) exposure in an ancestor leading to (2) molecular changes in germ cells, driving (3) a specific phenotype in descendants. However, convincing associations have been made between two of these three parameters, from a variety of diverse perspectives involving epidemiological, epigenetic, and genetic approaches (Fig. 1).

There are three general manners in which an ancestral exposure can be connected with a phenotype in descendants: (1) an exposure in an ancestor with a manifestation of a phenotype across multiple generations after the exposure was extinguished; this involves germline-based transmission as generations never exposed to the insult manifest the phenotype; this is often referred to as “transgenerational inheritance.” For females who are exposed in utero, the fetus (F1 generation) and fetal germ cells (which form the F2 generation) are directly exposed to the in utero insult; thus, F3 is the first generation that was not directly exposed and can be considered “transgenerational”. (2) Pre-fertilization exposure in a parent with the manifestation of a phenotype in children; in this instance, the parents, including the germ cell that will produce the offspring, were directly exposed; this does not demonstrate propagation through the germline independent of the exposure (this is often referred to as “intergenerational inheritance”). (3) Peri-fertilization exposure in an ancestor with the manifestation of a phenotype in the children; it is difficult in these cases to differentiate between pre-fertilization effects on the germline and direct in utero effects on the fetus. Indeed, the examination of events that occur around the time of fertilization reveals that this is a critical window in which exposures can affect the health of an individual throughout their life. These studies must be interpreted with caution, because there is a significant overlap with the developmental origins of the adult disease field, in which in utero, exposures program the fetus for diseases that do not manifest until adulthood. While the mechanism behind this effect is still epigenetic, it does not involve inheritance via germ cells. Nevertheless, these studies may detect transient effects on the germline that would not be detected if examined after time has passed.

The detection of non-equivalence between maternal and paternal alleles is also a powerful approach to detect non-genetic contributions to disease. Accumulating evidence from genome-wide association studies (GWAS) suggests that some genetic variants affect disease risk differently based on whether the allele was inherited from the mother or father.

### Challenges of studying non-genetic inheritance in humans

The study of transgenerational inheritance in humans is more complex than in model organisms for multiple reasons. In humans, the long time between initial exposure in ancestors and the manifestation of disease in descendants makes the discovery of a connection across generations particularly challenging, especially when this involves follow-up of families and examination of medical records over decades. In addition, many of the phenotypes examined in humans are complex diseases that are influenced by many factors that accumulate over one’s life with incremental effects, making studying the effect of any one single event on a single phenotype difficult. Similarly, the rigorously controlled experiments that are possible in inbred model organisms, in which the only difference is an ancestral exposure, are not practical in humans. As a result, in humans, this process is often studied based on an acute, profound event with a clear temporal onset in a well-defined population, and measurement of a clearly defined consequence (methylation of DNA, development of a disease) in descendants of the exposed group. Because these are not controlled experiments of a genetically homogenous population that only differs based on exposure, these studies are more prone to confounding effects, as discussed below. Despite these limitations, these studies have been invaluable in demonstrating the potential importance of non-genetic germline-driven inheritance in humans, supporting the need for further studies regarding the breadth of this process in disease pathogenesis, as well as the mechanism by which epigenetic abnormalities accumulate in germ cells and influence risk of disease in descendants.

This issue is further complicated by the genome-wide epigenetic reprogramming that occurs during mammalian germline and embryonic preimplantation development [12]. During this process, the majority of DNA methylation and histone modifications are erased, with a few exceptions at imprinted genes and retrotransposon sequences [13]. (Genomic imprinting is a process in which a gene is silenced based on whether it was inherited from the mother or the father [14]). Thus, it is unlikely that these epigenetic modifications by themselves are an adequate explanation for germline-mediated non-genetic inheritance of phenotypes. The persistence of some epigenetic marks during reprogramming, however, illustrates that some modifications can escape erasure and offers potential for the passage of epigenetic information across generations. While DNA methylation and histone modification marks are erased, there are other heritable factors that may persist, such as small RNA molecules [15].

### Confounders and critical considerations

For the reasons discussed above, there are numerous confounders that must be considered in human studies of non-genetic inheritance. They will be discussed generally here, and specific caveats will be mentioned below as applicable for specific exposures.

When examining changes in germ cell epigenetic molecules after an exposure, multiple potentially confounding factors must be considered. Exposures can affect the composition of cells within a population, influencing developmental timing, overall health, and survival, or the distribution of cell subsets within a population. As a result, differences in methylation may reflect global changes in the cell population, rather than a specific change in methylation within the cell. For example, when evaluating sperm, changes in DNA methylation may reflect alterations to sperm health or the percentage of motile sperm. In addition, there are factors associated with an exposure that is impossible to dissociate from that exposure and thus cannot be accounted for in a “control” population. For example, Shnorhavorian et al. demonstrated that adult males who received chemotherapy for cancer as adolescents had DNA methylation differences in their sperm when compared with a control population that never had cancer [16]. The authors concluded that exposure to chemotherapy leads to long-lasting epigenetic changes in the sperm. In this case, it must be considered that the environmental and/or genetic factors that predisposed these individuals to cancer also affected the sperm epigenome; also cancer itself may have affected the epigenetic profile of certain cell populations including germ cells. Another concern is that there are certain social and genetic factors that make some people more likely to be “exposed” than others, and there are sequelae of exposures that can change one’s life trajectory significantly, independent of any effect on heritable factors in germ cells. These wide-ranging effects are difficult to thoroughly understand and therefore very difficult to recapitulate in a “control” population. A further confounding factor is the underlying genetic variation in human populations; indeed polymorphisms in genomes may lead to stable alterations in epigenetic signatures independent of any exposures [17]; this is especially important when looking at epigenetic modifications at a large number of sites in a small number of individuals.

We currently have a poor understanding of the meaning and significance of epigenetic abnormalities detected in human germ cells [18–20]. Some studies report small changes in DNA methylation; despite statistical significance, the clinical significance of DNA methylation differences of a mild magnitude is unclear—that is, whether this has a meaningful effect on gene expression and subsequent phenotype. Similar questions arise for changes observed in regions of the genome that are not proximal to a gene. Also, for many of the reported methylation differences that localize in proximity to a gene, it is unclear whether these differences actually lead to a change in gene expression. A final consideration regarding the assessment of epigenetic factors in germ cells is whether observed differences are the consequence of a dedicated biologic pathway in which an exposure is translated into an epigenetic change. That is, do specific biologic pathways regulate this process based on specific exposures, or are the observed differences non-specific? It is also important to note that all of the epigenetic studies on human germ cells discussed here have been performed in males because sperm is obtained easier and in far greater numbers than oocytes. As a consequence, the role of female germline epigenetic factors in inheritance remains much less well described.

Confounding factors must also be considered when examining a connection between exposures in ancestors and phenotypes in descendants. In addition to germline-based inheritance, phenotypes can be transmitted across generations independent of the germline. One example is the transmission of behaviors, especially maternal care, across generations [21]. In these studies, germline-driven inheritance is excluded by cross-fostering, reinforcing the inheritance of some traits independent of the germline. Another example is the transmission of the microbiome across generations; the role of this phenomenon in disease inheritance in humans remains largely uncharacterized [22, 23].

The objective of this review is to examine the evidence for non-genetic germline-driven inheritance in humans. Despite the challenges in detecting an effect without the influence of multiple confounders, this mechanism of inheritance is a potentially significant contributor to human health and disease, and further investigation into its breadth and magnitude is of critical importance.

## Potential Examples of Non-Genetic Germline Inheritance in Humans

### Thyroid hormone

One of the most compelling examples of germline-based non-genetic inheritance involves fetal exposure to elevated thyroid hormone levels. Inhabitants of the Azorean island of Sao Manuel carry an autosomal dominant mutation in the thyroid hormone receptor β (THRβ) at high frequency. The heterozygous THRβ mutation manifests as a persistent elevation of serum thyroid hormone; in most instances, affected people do not have a significant hyperthyroid disease because this is a compensated hyperthyroid state without significant metabolic or organ disease. In cases where the mother has a heterozygous THRβ mutation, wild-type fetuses that did not inherit the mutation are exposed to high levels of thyroid hormone (TH) in utero, without being affected by sequelae of maternal hyperthyroid disease. (A critical caveat is that these fetuses are derived from oocytes that were heterozygous for this mutation before undergoing meiosis, and thus were exposed to the mutation. However, the exposure in this scenario is elevated blood thyroid hormone levels and not the mutation itself.)

The wild-type children of THRβ heterozygous mothers exhibit low birth weight, consistent with intrauterine exposure to high TH levels [24]. As adults, these wild-type individuals have impaired suppression of thyroid-stimulating hormone (TSH) in response to the administration of exogenous thyroid hormone, revealing an inherited reduced sensitivity to thyroid hormone (RSTH). Intriguingly, the children of F1 males (that is, males who were exposed to elevated TH in utero) exhibit evidence of RSTH, while the children of females did not exhibit this effect. The same pattern was observed in the F3 generation, whose great-grandmothers carried the THRβ mutation and whose grandfather was exposed to elevated TH in utero. Importantly, there was no difference in prolactin levels in these individuals, suggesting a specific pathway in which this phenotype is inherited in a manner independent of DNA mutations [24]. This is one of the clearest demonstrations that specific phenotypes can be inherited in response to specific ancestral exposures; in this case, the exposure is elevated levels of TH in utero. A similar phenomenon has been observed in mice, evaluating a different mutated gene and its role in early brain development in non-exposed descendants [25]. As discussed in the introduction, these compelling observations do not exclude a possible effect of non-germline factors contributing to the observed phenotype.

### Diet and exercise

The most well-known human examination of transgenerational inheritance comes from a series of studies examining a small town in Northeast Sweden known as Överkalix. This is a relatively isolated town where the food availability in the winter was dependent on the quality of that summer’s harvest. The people of Överkalix maintained detailed records of each year’s harvest as well as each citizen’s health, including longevity and cause of death. A close review of these records revealed that the quantity of grandparental food supply during the “slow growth period” of 8–12 years of age had a significant effect on the risk of mortality in their grandchildren, in a sex-specific manner. Specifically, the paternal grandfather’s food supply was associated with the mortality of grandsons but not granddaughters, and the paternal grandmother’s food supply was associated with the mortality of granddaughters only [26, 27]. An abundance of food during this time was associated with increased mortality in the descendants, and a scarcity of food was associated with decreased mortality in descendants. Further analysis revealed that a sharp change in the amount of food availability from more to less food or vice versa was associated with a transgenerational response only through the paternal grandmother; this effect was especially prominent in female grandchildren whose paternal grandmothers had a good harvest followed by a bad harvest [28]. Further examination revealed that the father’s poor food supply and mother’s good food supply were associated with decreased cardiovascular mortality in the F1 generation [27, 29]. These powerful studies took advantage of very detailed records over the decades and revealed a connection between grandparental food availability and risk of cardiovascular disease in grandchildren, with the transmission in a sex-specific manner. As with many other studies described here, the cellular and molecular processes that facilitate this process remain unknown, and the possible contribution by non-germline factors cannot be excluded.

A recent study that used historical records from the Uppsala Birth Cohort Multigenerational Study in Sweden replicated the Överkalix findings using a larger study cohort; the authors found that abundance of food prior to puberty in paternal grandfathers was associated with increased mortality in grandsons [30]. Interestingly, male grandchildren had a higher risk of dying from cancer (both tobacco-related and non-tobacco-related) if their paternal grandfather had good access to food prior to puberty. A similar study in China found that in utero exposure to famine increased the risk of type II diabetes in both the prenatally exposed individuals as well as their unexposed children (whose parents were exposed to the famine as a fetus). The increased risk of diabetes was strongest when both parents were exposed to famine in utero [31].

Examination of the Dutch Hunger Winter from 1944–1945 revealed that in utero exposure to famine was associated with increased risk of disease throughout life, including coronary artery disease, elevated cholesterol, altered clotting, and increased obesity [32, 33]. Furthermore, those exposed to famine in utero had decreased cognitive ability in their 6th decade of life [34]. Examination of DNA methylation of the gene IGF2, an imprinted gene critical for in utero growth and development, in whole blood of individuals exposed to famine in utero revealed decreased methylation when compared with unexposed same-sex siblings [35]. In addition, the children of prenatally undernourished fathers had increased adiposity and BMI [36]. It is important to remember that in utero effects may account for much of these observed effects.

Chewing betel nut (*Areca catechu*) is a common habit and cultural tradition in Southeast Asia; regular use can cause serious health effects. An observational study in Taiwan found that offspring of fathers who practiced betel nut chewing, after controlling for the presence of maternal and paternal metabolic syndrome and the habit of chewing betel nut themselves, had a 2.5-fold increase in the risk of the early development of metabolic syndrome compared with those who were not exposed [37].

Exercise and weight change have been shown to alter the epigenetic profile of sperm. Individuals who underwent 6 weeks of endurance training demonstrated changes in DNA methylation and small RNA expression, especially piRNAs. Gene ontology analysis of the differentially methylated regions revealed enrichment around genes that regulate neurologic function [38]. Importantly, the sites of aberrant methylation did not overlap with DNA methylation changes detected in a different examination of endurance training [39]. In a similar study, surgery-induced weight loss from gastric bypass was associated with a change in sperm DNA methylation, especially around genes that regulate appetite [40].

### Stress

The traumatic experience of a parent can have significant effects on their offspring, potentially from inheritance through the germ cells [41, 42]. The effect of trauma on phenotypes across generations is especially difficult to study because of the confounding influence of altered parenting that may distort any phenotypic or epigenetic evaluation. The most well-known study of this type found that the adult offspring of Holocaust survivors had decreased DNA methylation of the gene FK506-binding protein 5 (FKBP5) in the blood; FKBP5 is an important regulator of glucocorticoid receptor sensitivity and therefore is critical in the response to stress. These findings persisted after controlling for parental post-traumatic stress disorder (PTSD) as well as a polymorphic variant associated with altered stress response; the authors conclude that parental Holocaust exposure was a significant predictor of offspring FKBP5 methylation in this group [43]. These methylation differences are functionally relevant, as the degree of methylation at this site was associated with levels of the stress hormone cortisol. This finding was supported by a different study from the same group that found altered cortisol metabolism in offspring of Holocaust exposed mothers [44]. The effects of altered parental care in Holocaust survivors is a potential significant confounding factor in these intriguing reports, and these findings must be interpreted cautiously without overinterpretation of the findings or their larger societal implications [45]. The same group also found lower salivary cortisol levels in the 1-year-old offspring of mothers with PTSD after the World Trade Center collapsed during their pregnancy [46]; lower salivary cortisol is indeed linked to increased vulnerability to PTSD [47].

The effect of parental stress on descendants was further supported in a study that examined DNA methylation in individuals whose grandmothers reported interpersonal violence during pregnancy, which was used as a surrogate for stress. Examination of the cells found in the saliva of these individuals revealed DNA methylation abnormalities in four genes associated with cardiovascular processes [48]. Taking a different approach involving the examination of historical records, Costa et al. found that the sons of Civil War ex-prisoners of war (POWs) who were imprisoned when camp conditions were at their worst were 1.11 times more likely to die than sons of non-POWs, while no impact of paternal ex-POW status was observed in daughters [49]. In all of these studies, it is important to appreciate that the primary exposure has widespread effects that generate multiple confounders that must be considered when evaluating the conclusions.

Individuals conceived during the 1918 influenza pandemic had a higher frequency of physical and socioeconomic sequelae such as physical disability, decreased educational attainment, and lower income, when compared with a similar cohort of individuals not conceived during the pandemic [50]. Of note, this study did not differentiate those individuals whose mothers were infected; thus, the effect seems to be caused by the stressors associated with living through a pandemic rather than a direct consequence of exposure to a virus. Mazumder et al. showed that prenatal exposure to the 1918 influenza pandemic was associated with ≥20% excess ischemic heart disease after the age of 60 years [51]. These studies do not clearly distinguish between inheritance mediated through the germline as opposed to a direct in utero effect.

Examination of sperm epigenetic factors has revealed abnormalities associated with stressful life experiences. Childhood abuse was associated with significant changes in sperm DNA methylation, especially around genes that regulate neuronal function, adipose cell function, and the immune system [52]. In addition, early life stress during childhood was associated with a decrease in sperm miRNAs implicated in brain development, stress regulation, and spermatogenesis [53]. These findings were validated in a mouse model of stress; how these changes affect gene expression and subsequent phenotypes remains unclear. Because the gene targets of these miRNAs are not well defined, it remains unclear whether there is overlap in the genes targeted by epigenetic modifications in these two studies.

### Smoking

Maternal and paternal cigarette smoking has been associated with inheritance of phenotypes in children and grandchildren. Women who smoked while pregnant had grandchildren with an increased risk of asthma [54, 55]. Grandmother’s smoking during pregnancy was also associated with asthma in their daughters and asthma with nasal allergies in their grandchildren through the maternal line, irrespective of the mother’s smoking status or maternal asthma [56]. Similarly, paternal smoking during adolescence independently increased the risk of childhood asthma in offspring. A longitudinal multigenerational study in northern Europe found that non-allergic asthma was more common in offspring of fathers who smoked before conception, and the risk was highest if the father started smoking before 15 years of age [57]. Grandmaternal smoking has also been associated with autism in granddaughters; this effect was strongest if the mother did not smoke during pregnancy [58]. Evidence from these multi-generational studies suggests that tobacco smoking causes inheritable modifications that increase the risk of asthma in future generations. However, a critical consideration is that cigarette smoke is a human germ cell mutagen [59], adding an additional confounder to these findings.

Studies on germ cell epigenetic factors revealed that the sperm of smokers had altered miRNA expression, especially those miRNAs that regulate pathways essential for sperm and embryo development [60]. As discussed above, it is possible that smoking has a global effect on sperm development and survival, which could explain these findings. From a different perspective, males who regularly smoked cannabis had changes in sperm DNA methylation over approximately 4000 DNA methylation sites. The same group observed significant hypomethylation in cannabis smokers around the gene DLGAP2, which controls neuronal synapse organization and has been associated with autism [61]. The authors show that there is a >20% methylation difference across 9 different sites of methylation of DLGAP2; they also show that increased methylation at these sites leads to decreased expression of the DLGAP2 gene.

### Chemicals

There is strong evidence in mouse and rat studies that endocrine-disrupting chemicals modify germ cell epigenetic processes and influence phenotypes across multiple generations [62, 63]. In humans, women prenatally exposed to the endocrine-disrupting chemical diethylstilbestrol (DES) have an increased risk of reproductive tract anomalies, menstrual irregularities, infertility, pregnancy loss, and vaginal adenocarcinoma [64]. F2 women (daughters born to prenatally DES-exposed mothers) have been found to attain menstrual regularization later; they also were more likely to report irregular menstrual periods [65, 66]. Similarly, F2 males (sons born to prenatally DES-exposed mothers) had an increased risk of hypospadias and testicular germ cell tumors [67–69]. A recent study by Kioumourtzoglou et al. found that prenatal exposure to DES during the first trimester of pregnancy was associated with higher rates of attention-deficit/hyperactivity disorder (ADHD) in third-generation men and women [70]. DES has effects on estrogen signaling and exposure to DES has been shown to affect DNA methylation in germ cells [71]. Again, these observed associations do not exclude the role of non-germline factors.

Exposure to lead causes multi-organ abnormalities, including memory loss, difficulty concentrating, elevated blood pressure, and kidney injury. Newborns from women who had high blood lead levels as neonates themselves had abnormal DNA methylation at more than 500 loci around genes that control processes such as spatial learning, memory, metabolic diseases, and autoimmune disorders. It is important to recognize that the DNA was measured in blood, and germ cells were not analyzed. Interestingly, the authors found that the altered DNA methylation profiles of the grandchildren’s blood normalized during post-natal development [72].

Exposure to the chemical warfare molecule “agent orange” has been associated with spina bifida in the offspring of exposed males [73]. A recent examination of frozen semen in those with high exposure to this chemical during the Vietnam War revealed DNA methylation abnormalities at specific genomic sites [74].

Males with higher concentrations of urinary organophosphate metabolites, which originate from flame-retardants, had significantly higher fractions of aberrantly methylated DNA in sperm at differentially methylated regions (DMRs) of multiple imprinted genes [75]. Various environmental exposures, including mercury [76] and polycyclic aromatic hydrocarbons [77] were associated with mild DNA methylation changes at imprinted genes in sperm; again, it is unclear the significance of these changes on affecting phenotypes or disease risk. Environmental exposure to bisphenol A, a chemical found in many plastics, was associated with changes in sperm hydroxymethylation, which is an intermediate modification in the process of DNA demethylation and is associated with active gene expression [78]**.**

### Periconceptual cold exposure

A retrospective study of over 8000 humans revealed a connection between being conceived in a cold season and metabolic changes in brown adipose tissue activity [79]. Brown adipose tissue is hyperactive and associated with lower body mass index (BMI), leading the authors to speculate that periconceptual cold exposure improves metabolism and protects from diet-induced obesity. This finding was supported in mice, where paternal perinatal cold exposure recapitulates the phenomenon described in humans and was associated with gene expression changes in brown adipose tissue that could explain this observation; the authors also found changes in the sperm DNA methylation of cold-exposed males that could explain altered metabolic gene expression [79].

### Sperm methylation and risk of autism in children

Examination of sperm DNA methylation in fathers of autistic children revealed significant differences around genes that regulate developmental processes, including the C/D box small nucleolar RNA (SNORD) locus associated with the imprinting disorder Prader-Willi Syndrome [80]. This is the only study we discovered that examined germ cells for epigenetic abnormalities in fathers whose children have a diagnosed disorder (that is not an “epigenetic disorder”).

### Non-equivalence of maternal vs. paternal alleles

Historically, most genetic studies assume the paternally-inherited and maternally-inherited alleles are identical if they contain the same DNA sequence. However, there is accumulating evidence that these alleles are not interchangeable, and inheritance of the same allele can have a varying effect on disease depending on whether it came from the mother or the father, despite having identical DNA sequences. This perspective provides a unique insight into the role of non-genetic inherited factors in disease development.

One of the most striking examples of this phenomenon is the observation that congenital heart disease (CHD) occurs more frequently in children of mothers with a history of CHD when compared with children of fathers with a history of CHD [81]. Further examination of this phenomenon revealed that this pattern is most clear for certain CHDs such as pulmonic stenosis or aortic coarctation [82]. The cellular and molecular mechanisms behind this process remain unknown, and the current knowledge does not exclude non-germline mediated forms of inheritance. For example, it is possible that the intrauterine environment of mothers with a history of CHD predisposes to abnormalities of early heart development.

A study performed in Iceland examined whether single nucleotide polymorphisms (SNPs) within 500 kilobases of an imprinted gene had differential effects on disease risk depending on whether they were paternally—or maternally-inherited. In this study, a parent-of-origin association was discovered between specific SNPs and breast cancer, basal cell carcinoma, and type II diabetes [83]. The study was designed to specifically examine these regions around imprinted genes; examination of larger parts of the genome may reveal many more associations.

Similar parent-of-origin effects (POE) were described in Hutterites, a founder population of European descent. A total of 21 common disease-associated phenotypes were examined in a single large pedigree, and the authors found evidence of a POE in 11 of these phenotypes, especially traits that have an effect on cardiovascular health. They also discovered some loci that have opposing effects depending on whether an allele was inherited from the mother or father. Some of these loci had characteristics similar to known imprinted genes and were associated with the expression of nearby genes [84].

A GWAS performed to discover SNPs associated with esotropia (turning-in of one or both eyes) revealed an SNP near an imprinted locus [85]. Further analysis revealed that the inheritance of the SNP on the non-methylated paternal allele was associated with a significantly increased risk of esotropia compared with the inheritance of the maternal allele, where the SNP is in close proximity to the methylated allele.

POE have been described in congenital heart defects [86], attention deficit-hyperactivity disorder [87], testicular germ cell tumors [88], cleft lip [89], autism [90, 91], language impairment [92], type II diabetes [93, 94], adiposity [95], and BMI [96]. Some of these SNPs are located in proximity to imprinted genes; further evaluation is needed to determine whether all of these effects are mediated by the ~100 imprinted genes in the human genome, or whether there are additional processes that affect asymmetry in the maternal and paternal genomes.

## Conclusion

This review summarizes the evidence for the inheritance of phenotypes and diseases in humans through the germline via non-genetic mechanisms. The data comes from a variety of sources, including epidemiological, genetic, and epigenetic studies. For many of these examples, there are alternative explanations for the observed associations; however, when taken collectively, these studies suggest that there is more to inheritance through the germline than what is encoded by the DNA in sperm and oocytes.

Because this line of investigation into human diseases is in its infancy, it is very possible that there are exposures that predispose to significant diseases that have yet to be discovered. Indeed, there are many diseases with evidence of a strong inherited component, yet an absence of known causative DNA mutations [97–99]. As discussed throughout this review, the detection of non-genetic inheritance in humans is technically difficult, both in terms of connecting exposures to meaningful molecular abnormalities in germ cells, as well as detecting an ancestral exposure that correlates with a descendant’s diseases. We are currently at the stage of observing correlations between exposures and diseases. These studies often start with an exposure, followed by an examination of the germ cells of those exposed, or the phenotypes of their descendants.

Moving forward, continued support of this phenomenon in humans requires consideration of the myriad of potential confounding factors when designing and executing experiments. In this manner, prospective studies can provide invaluable insight into germline non-genetic inheritance; the practical drawback of this approach is the long latency time between exposure and manifestation of a phenotype across generations in humans. In addition, it will be imperative to assess, empirically quantify, and correct for genetic effects on epigenetic variation, to the extent that this information is known and can be applied to the experimental approach. Continued advancement of this field will also be dependent on linking observed epigenetic changes with specific phenotypes.

Future studies may benefit from focusing on a specific disease with evidence of strong heritability that cannot be explained by changes in the DNA sequence and working backward to examine ancestors’ exposures and germ cells. For example, roughly 60% of congenital heart disease cases that run in families across more than one generation do not have a known genetic cause [100]. A focused investigation into ancestors’ life history may reveal enrichment for a previously ignored exposure. Similarly, sperm from the father of an affected individual can be examined for abnormalities in DNA methylation and small RNA expression. However, it must be remembered that the single “fertilizing” sperm may have been aberrant compared with the other sperm from that individual; in this case, the assessment of epigenetic abnormalities averaged over many sperm may miss a rare, epigenetically abnormal sperm. Another very important limitation is that it is currently not possible to regularly obtain and examine oocytes, so disease risk that is passed through the maternal line is missed. In sum, ancestral exposures may affect the risk of a number of diseases in descendants; however, any examination of this phenomenon in humans must consider the challenges in detecting a connection between exposure and disease and appreciate the significant confounding factors that can influence such an association.

## Data Availability

Not applicable.

## Abbreviations

GWAS: Genome-wide association studies
THRβ: Thyroid hormone receptor β
TH: Thyroid hormone
TSH: Thyroid-stimulating hormone
RSTH: Reduced sensitivity to thyroid hormone
IGF2: Insulin-like growth factor 2
FKBP5: FK506-binding protein 5
PTSD: Post-traumatic stress disorder
POWs: Prisoners of war
DLGAP2: Discs large homolog-associated protein 2
DES: Diethylstilbestrol
ADHD: Attention-deficit/hyperactivity disorder
DMRs: Differentially methylated regions
BMI: Body mass index
SNORD: C/D box small nucleolar RNA
CHD: Congenital heart disease
SNPs: Single-nucleotide polymorphisms
POE: Parent-of-origin effects
